# Uremic Conditions Drive Human Monocytes to Pro-Atherogenic Differentiation via an Angiotensin-Dependent Mechanism

**DOI:** 10.1371/journal.pone.0102137

**Published:** 2014-07-08

**Authors:** Bogusz Trojanowicz, Christof Ulrich, Eric Seibert, Roman Fiedler, Matthias Girndt

**Affiliations:** Department of Internal Medicine II, Martin-Luther-University Halle-Wittenberg, Germany; Maastricht University Faculty of Health, Medicine, and Life Sciences, Netherlands

## Abstract

**Aims:**

Elevated expression levels of monocytic-ACE have been found in haemodialysis patients. They are not only epidemiologically linked with increased mortality and cardiovascular disease, but may also directly participate in the initial steps of atherosclerosis. To further address this question we tested the role of monocytic-ACE in promotion of atherosclerotic events in vitro under conditions mimicking those of chronic renal failure.

**Methods and Results:**

Treatment of human primary monocytes or THP-1 cells with uremic serum as well as PMA-induced differentiation led to significantly up-regulated expression of ACE, further increased by additional treatment with LPS. Functionally, these monocytes revealed significantly increased adhesion and transmigration through endothelial monolayers. Overexpression of ACE in transfected monocytes or THP-1 cells led to development of more differentiated, macrophage-like phenotype with up-regulated expression of Arg1, MCSF, MCP-1 and CCR2. Expression of pro-inflammatory cytokines TNFa and IL-6 were also noticeably up-regulated. ACE overexpression resulted in significantly increased adhesion and transmigration properties. Transcriptional screening of ACE-overexpressing monocytes revealed noticeably increased expression of Angiotensin II receptors and adhesion- as well as atherosclerosis-related ICAM-1 and VCAM1. Inhibition of monocyte ACE or AngII-receptor signalling led to decreased adhesion potential of ACE-overexpressing cells.

**Conclusions:**

Taken together, these data demonstrate that uremia induced expression of monocytic-ACE mediates the development of highly pro-atherogenic cells via an AngII-dependent mechanism.

## Introduction

Systemic chronic inflammation plays a crucial role in the initiation and progression of atherosclerosis. Patients with chronic kidney disease (CKD) maintained on intermittent hemodialysis develop progressive atherosclerosis leading to cardiovascular events such as myocardial infarction and stroke [1], [2], [3]. These patients are at higher risk of all-cause mortality [4] and obviously suffer from both atherosclerosis [5] and arteriosclerosis [6]. Recent data suggest that inflammation is predominantly associated with that plaque forming disease of atherosclerosis rather than vascular stiffening [7].

So called “classical” cardiovascular risk factors such as dyslipidemia, hypertension, diabetes or smoking promote initiation and progression of atherosclerosis by recruitment of circulating immune cells to infiltrate the injured vascular endothelium [8], [9]. The monocytes infiltrating in the subendothelial space differentiate into macrophages and dendritic cells which trigger the accumulation of lipids, extracellular matrix components and other cells in the vessel wall. Extensive and prolonged accumulation of lipid-carrying apoptotic cells, cell debris and cholesterol crystals leads to the formation of atherosclerotic plaque [10]. Infiltrating monocytes may differentiate into different macrophage subtypes with either protective or pathogenic activities. Recent studies suggest that classically activated macrophages (M1 or CAMs) may possess pro-atherogenic abilities in contrast to alternatively activated athero-protective macrophages M2 (or AAMs) [11], [12]. However in advanced stages of atherosclerosis, secretion of metalloproteinases, typical for M2-like macrophages, may contribute to matrix degradation and rupture, which may trigger a myocardial infarction or stroke [13], [14], [15], [16].

Angiotensin converting enzyme (ACE) participates in the regulation of blood pressure (arterial vasoconstriction) and sodium and water balance. The circulating exopeptidase catalyzes the conversion of decapeptide angiotensin I (AngI) to octapeptide angiotensin II (AngII), a potent vasoconstrictor. ACE is not only secreted by pulmonary and renal endothelial cells, but is also expressed on the surface of monocytes, macrophages and smooth muscle cells [17]. Furthermore, ACE has also been detected in human atherosclerotic lesions, where it is associated with a subset of macrophages [18], [19], [20] or cells possessing dendritic-like properties [21].

The presence of angiotensinogen (AGT), AngI, AngII and its receptors on human monocytes indicate that this cell type might have a functionally relevant auto- or paracrine angiotensin system potentially involved in the pathogenesis of vascular disease [22]. Inhibition of ACE and/or employment of AngII receptor 1 (AT1R) antagonists has been shown to be effective in decreasing clinical events in patients with atherosclerosis [23]. Little is known about local cellular effects besides systemic blood pressure control, particularly on monocytic ACE (mACE).

In patients with end stage renal disease circulating monocytes are activated and proinflammatory monocytes are expanded. We recently demonstrated that elevated levels of mACE on those cells in hemodialysis patients are associated with increased mortality and cardiovascular morbidity and may also participate in the initial steps of atherosclerosis [7], [24], [25].

In this study we used the primary human monocytes and myelomonocytic cell line THP-1 to investigate the regulation of mACE under conditions of uremia. Furthermore, a potentially causal involvement of ACE upregulation for atherosclerosis initiation was studied.

## Materials and Methods

### Human serum and isolation of primary human monocytes

Pooled uremic sera (HD) were obtained from chronic hemodialysis patients (n = 10; both males and females) treated at the Dialysis Ward of the University Clinic Halle. All HD patients were treated three times weekly by standard bicarbonate hemodialysis with ultrapure water quality (by reverse osmosis and sterile filters) using high flux polynephron membranes (Nipro Europe). All HD samples were obtained prior to a regular hemodialysis session from the dialysis access and anti-coagulated with heparin. Pooled uremic sera from peritoneal dialysis (PD) were obtained from 5 patients (both males and females). Additionally pooled uremic sera were isolated from 3 CKD5 patients (males) not on dialysis and 10 HD patients (both males and females) prior to (pre-HD) and after (post-HD) a hemodialysis session. HD, PD, CKD 5, pre- and post-HD patients were aged between 40 and 70 years, they were free of acute infectious complications and were not treated by immunosuppressive medication. Normal sera (NS) were obtained from age matched healthy volunteers (n = 8; age range 50 to 67 years; both males and females). Human primary monocytes were isolated from 3 healthy volunteers by employment of Pan Monocyte Isolation Kit (Miltenyi) and AutoMacs cell separator (Miltenyi) according to manufacturer's instructions. All healthy volunteers were normotensive and none was taking any medication. The isolation of the human sera and primary monocytes were performed according to the declaration of Helsinki. This study was approved by the ethical committee of the Martin Luther University, Faculty of Medicine. All patients and persons involved in this study gave written consent.

### Cell culture and treatments of primary human monocytes and THP-1 cells

THP-1 monocytes were cultured in RPMI (Sigma) medium, supplemented with 1.125 g/l sodium carbonate, 10% heat inactivated (65°C/30 min) fetal calf serum (FCS) and 100 µg/ml penicillin/streptomycin (Biochrom). THP-1 cells (1×10^6^/well) cells were treated with RPMI medium containing 10% HD or NS or 10 ng/ml LPS (Sigma) or combination of LPS and human sera in the presence of 10 ng/ml PMA (Sigma) as differentiating agent for 72 h. Treatment medium was replaced daily.

3×10^5^/well primary human monocytes were treated with 10% NS or HD or PD or CKD5 or pre-HD or post-HD sera for 72 h in RPMI medium without FCS. All treatments were performed in hydrophobic 6-well plates (Greiner bio One) in a standard humidified incubator (37 °C, 5% CO2). Total RNA from THP-1 cells and human primary monocytes was isolated at 0 h, 24 h, 48 h and 72 h by employment of Tri Reagent (Sigma) and ZR RNA MiniPrep Kit (Zymo Research), respectively, both according to manufacturer's instructions.

ACE/AngII-receptor inhibition studies were performed with captopril (ACE inhibitor, Santa Cruz Biotechnology) or losartan (Angiotensin II type 1 receptor (AT1R) antagonist, Santa Cruz Biotechnology) or angiotensin II (Sigma). Briefly calcein-labelled (calcein-AM, 1 µM, Cayman Chemical) control or ACE-overexpressing monocytes were tested for their adhesion abilities to endothelial monolayers in the presence or absence of 500 nM captopril or 1 µM losartan for 30 min. Additionally endothelial-adhesion of ACE-negative THP-1 cells was tested in the presence or absence of Angiotensin II (1 µM) or losartan (1 µM). For details see below (adhesion assay) and corresponding figure legends.

HUVEC (Human umbilical vein endothelial cells) cell line was cultured in Medium 200 supplemented with LSGS (Low Serum Growth Supplement, Gibco).

### Transfection

For generation of THP-1 cells stably overexpressing human ACE, the cells were transfected with pcDNA3.1(-) carrying the full coding sequence of ACE (a gift from Dr. K. Kohlstedt, Goethe-University, Frankfurt am Main, Germany). Control cells received empty plasmid only. Transfection of the cells was performed in 24-well plates in normal growth medium. 1×10^6^ THP-1 cells/well were transfected with 1 µg of ACE or empty plasmid in the presence of Lipofectamine 2000 as a carrier. After 24 h the cells were selected with growth medium supplemented with 1000 µg/ml neomycin G-418 (Biochrom) for next 8 weeks. Thereafter, the concentration of neomycin in growth medium was set up to 500 µg/ml. Transient transfection of primary human monocytes was performed with the same plasmids in 24-well plates in RPMI medium without FCS. 1×10^6^/well of pooled primary human monocytes, obtained from volunteers were transfected with 1 µg of ACE or empty plasmid and Lipofectamine 2000 in the presence of RPMI medium. Expression of ACE and corresponding assays were performed 24 h after transfection.

### RNA isolation and Real Time PCR

Total RNA was isolated using Tri-Reagent (Sigma) or ZR RNA MiniPrep Kit (Zymo Research), both according to manufacturer's instructions. Depending on the experiment 50 ng-1 µg of total RNA was used as a template for first strand cDNA synthesis employing High Capacity cDNA Reverse Transcription Kit according to manufacturer's instructions (Life Technologies). The samples were stored at -20°C.

Amplifications of ACE, TNFa, IL-6, 18S and RPL37A were performed with TaqMan Gene Expression Assays (Life Technologies) and FastStart Universal Probe Master Mix (Roche) in a StepOne plus System. 18S and RPL37A (Ribosomal Protein 37a) were employed for normalization of target mRNA expression. Thermal cycling conditions were as follows: hold 10 min at 95°C, followed by 40 cycles of 10 sec at 95°C and 30 sec at 60°C. Amplifications of, MCSF (Macrophage Colony-Stimulating Factor), AT1R (Angiotensin II Type I Receptor), AT2R (Angiotensin II Type II Receptor), ICAM-1 (Intercellular Adhesion Molecule 1), VCAM-1 (Vascular Cell Adhesion Molecule 1), MCP-1/CCL2 (Monocyte Chemotactic Protein-1), Arg1 (Arginase-1), Arg2 (Arginase-2), iNOS (inducible nitric oxide synthase) and RPL37A were performed with sequences presented in Table 1 and Maxima SYBR Green-qPCR Master Mix (Thermo Scientific). RPL37A was employed for normalization. Thermal cycling conditions were as follows: hold 10 min at 95°C, followed by 40 cycles of 15 sec at 95°C, 30 sec at 60°C and 30 sec at 72°C. Data evaluation was performed with DataAssist Software (Life Technologies).

**Table 1:** Primer sequences employed in this study.

Target	Sequences
RPL37A	S-5′-ATTGAAATCAGCCAGCACGC, AS-5′-AGGAACCACAGTGCCAGATCC
MCSF	S-5′-TAGCCACATGATTGGGAGTG, AS-5′-TATCTCTGAAGCGCATGGTG
AT1R	S-5′-GCACAATGCTTGTAGCCAAA, AS-5′-GGGTTGAATTTTGGGACTCA
AT2R	S-5′-TTCCCTTCCATGTTCTGACC, AS-5′-AAACACACTGCGGAGCTTCT
ICAM-1	S-5′-GGCCTCAGTCAGTGTGA, AS-5′-AACCCCATTCAGCGTCA
VCAM-1	S- 5‘-CCGGATTGCTGCTCAGATTGGA, AS- 5‘-AGCGTGGAATTGGTCCCCTCA
MCP-1	S-5′-TCGCGAGCTATAGAAGAATCA, AS-5′-TGTTCAAGTCTTCGGAGTTTG
Arg1	S-5′-GGAGACCACAGTTTGGCAAT, AS-5′-CCACTTGTGGTTGTCAGTGG
Arg2	S-5′-TTCCATCCTGAAGAAATCCG, AS-5′-AGAGCCTTTTCATCAAGCCA
iNOS	S-5′-AAAGACCAGGCTGTCGTTGA, AS-5′-ACGGGACCGGTATTCATTCT

### MTT

In 96-well plates, 3000 THP-1 cells/well were seeded and cultured in growth medium for 24h, 48h and 72 h. For MTT assay, cells were then stained with MTT (3-[4,5-dimethylthiazol-2-yl]-2,5-diphenyltetrazolium bromide, Sigma) for 4 h at 37 °C and shortly incubated with dimethyl sulfoxide (DMSO, Roth). Thereby, a coloured formazan salt develops depending on the availability of mitochondrial NADH2 only in living, but not dead cells. Optical density was measured with BioRad ELISA reader at 550 nm. Experiment was repeated at least three times.

### Attachment assay

In 96-well plates, 3000 THP-1 cells/well were seeded and cultured in growth medium in the presence of 10 ng/ml PMA for 72 h. Thereafter the cells were fixed with 4% PFA/PBS for 30 min and vigorously washed with PBS. Remaining cells were stained with 0.2% crystal violet (CV, dissolved in water and filtered before use, Roth) for 30 min at RT. The excess of CV was removed by washing with deionized water until no violet colour release from culture plate was observed. Cellular CV was solubilized by addition of 0.1% SDS (Roth). The absorbance was measured after 15 min. incubation at RT at 550 nm. In long time attachment experiments 1 × 10^6^ THP-1 cells were incubated in the presence of 10% NS or HD sera for 72 h. After two times washing with PBS, adhered cells were detached with Accutase and counted with FACS. Experiment was repeated at least three times.

### Adhesion assay

Adhesion of primary human monocytes or THP-1 cells to endothelial HUVEC monolayers was investigated in 24-well plates. Briefly, HUVEC cells were cultured in growth medium until they created monolayer on the bottom of 24-well plate. For labelling, primary monocytes or THP-1 cells were incubated in RPMI containing 1 µM calcein-AM at 37 °C for 60 min. The cells were washed with RPMI twice. Depending on the experiment fluorescence-labelled cells were re-suspended in 2 ml RPMI medium or RPMI medium containing 10% NS or HD or PD or CKD5 sera. HUVEC monolayers were washed twice with RPMI medium before addition of 2 ml labelled primary monocytes (3×10^5^ cells/well) or THP-1 cells (1 × 10^6^ cells/well) per well. The plates were incubated for 30 min at 37 °C. After incubation, the monolayer was gently washed three times with RPMI. Adherent monocytes were photographed using a Biozero BZ-9000 fluorescence microscope (Keyence). The number of the adherent cells was evaluated in 10 microscopic fields for each situation by employment of ImageJ software (Wayne Rasband, National Institutes of Health, USA). All experiments were repeated at least three times.

### Transmigration assay

All transmigration assays were performed by employment of Millicell Cell Culture Inserts (Millipore, 8 µM pore size, 12 mm diameter). Primary monocytes or THP-1 cells were incubated in RPMI medium containing 1 µM calcein-AM at 37 °C for 60 min. The cells were washed with RPMI twice. 3×10^5^ primary monocytes or 5×10^5^ calcein-labelled THP-1 cells were re-suspended in 400 µl RPMI medium and seeded into upper transmigration chamber. Depending on the experiment, the cells transmigrated through uncoated or HUVEC monolayers coated membranes towards lower chamber filled with 600 µl RPMI medium supplemented with 50 ng/ml MCP-1 (Miltenyi) or 10% NS or HD or PD or CKD5 sera. In some experiments, THP-1 cells transmigrated towards HUVEC monolayers created on the bottom of lower chamber in the presence of RPMI only in both chambers. All transmigrations were performed at 37 °C for 60 min in the absence of FCS. Transmigrated cells were visualized by fluorescence microscopy (Biozero BZ-9000, Keyence) and counted in 10 microscopic fields for each situation by employment of ImageJ software (Wayne Rasband, National Institutes of Health, USA). In some experiments transmigrated cells were counted by FACS. Experiment was repeated at least three times.

### Immunocytochemistry

1×10^5^ THP-1 cells were seeded in chamber slides and let grown in normal medium for 24 h. The cells were gently washed with PBS and fixed in 4% PFA/PBS mixture for 10 min at RT. After washing twice with PBS, cells were incubated overnight with FITC-labelled mouse anti-human ACE antibody (AbD Serotec, clone 9B9, diluted 1.100 with PBS) at 4°C in the dark. Negative control was exposed to FITC-labelled IgG2a mouse anti-human antibody (BD Pharmingen) and processed as described above. After 3×10 min washing in PBS, the slides were covered with mounting medium containing a DAPI dye (Vectashield, Vector Laboratories) and dried 1 hour at RT in the dark. Microscopic investigations were performed with fluorescence microscope (Biozero BZ-9000, Keyence). Additionally the living cells were photographed in a phase contrast modus with the same microscope.

### FACS

5×10^4^ cells were washed twice with MACS buffer (1× PBS supplemented with 0.5%BSA 2 mM EDTA and 0.07% NaN3) and incubated with FITC-conjugated mouse anti-human ACE (AbD Serotec, clone 9B9) and PE-conjugated mouse anti-human CCR2 (R&D, clone 48607) for 30 min at RT in the dark. Measurements were performed with MacsQuant (Miltenyi) flow cytometer. Expression data (Isotype corrected) were presented as mean fluorescence intensity (MFI).

### Statistics

Each experiment was repeated at least three times. Data are presented as mean ±SD. Statistical significance was calculated by employment of two-sided Student-t-test. The values of p<0.05 were considered statistically significant. Bonferroni correction was applied for multiply comparisons dividing the significance level by the number of tested variables.

## Results

### Uremic serum or LPS increase the expression of ACE

**Figure 1:**
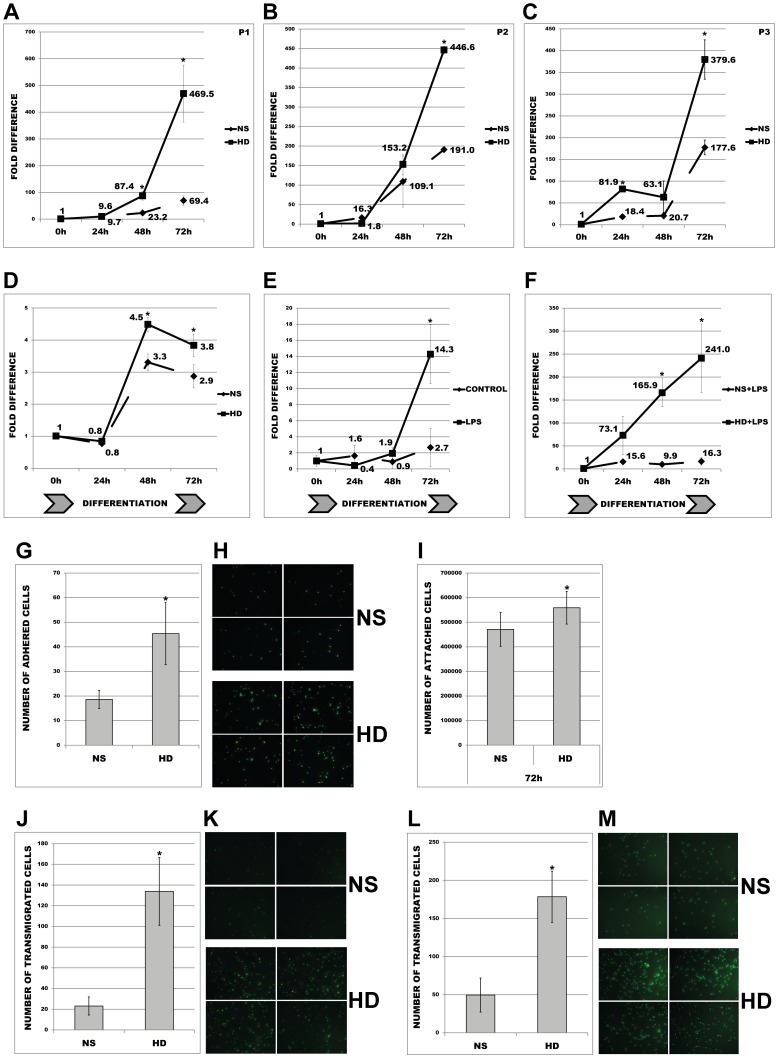
Expression of ACE and behaviour of human monocytes under uremic status I. (A, B, C) Primary human monocytes obtained from healthy volunteers P1, P2, and P3 were treated with 10% NS or HD sera for 72 h and investigated for ACE-mRNA expression. Means ± SD of three independent experiments. (D, E, F) THP-1 monocytes were PMA-differentiated (10 ng/ml) into macrophages in the presence of (D) 10% NS or HD sera or (E) 10 ng/ml LPS or (F) 10 ng/ml LPS and both of them for 72 h. Means ± SD of three independent experiments. (G, H, I) Attachment and adhesion of THP-1 monocytes and primary monocytes treated with NS or HD. Primary human monocytes were incubated in the presence of 10% NS or HD sera for 30 min and investigated for their endothelial-adhesion. The number (G) and corresponding images of endothelial-adhered monocytes (H) are shown. Means ± SD of cell number in 10 microscopic fields in three independent experiments. (I) THP-1 were incubated in the presence of 10% NS or HD sera for 72 h and investigated for their attachment abilities. The number of attached cells (detached prior to counting) was counted by FACS. Means ± SD of four independent experiments. (J, K, L, M) Transmigration of calcein-labelled primary monocytes (J, K) or THP-1 cells (L, M) through endothelial monolayer towards lower chamber filled with RPMI medium supplemented with 10% NS or HD sera. Transmigrated cells were visualized with fluorescent microscopy (K, M) and counted in 10 random fields (J, L). Representative images are shown. Means ± SD of cell number in 10 microscopic fields in three independent experiments. * p< 0.05 indicates statistical significance.

To test whether uremic serum or inflammatory conditions are able to regulate the expression of ACE we subjected primary human monocytes or undifferentiated THP-1 cells to 72 h treatment with normal (NS) or uremic (HD) serum or LPS. As shown in Fig. 1A–C primary human monocytes (P1, P2, P3) treated with HD revealed dramatically increased expression of ACE resulting after 72 h in an up-regulation range of 379.6–469.5 fold in all investigated samples. In order to investigate the uremic regulation of ACE during PMA-mediated differentiation into macrophages in the presence of HD or inflammatory conditions, THP-1 were incubated with 10 ng/ml PMA and corresponding sera or 10 ng/ml LPS or both of them for 72 h. As demonstrated in Fig. 1D, the cells differentiated in uremic conditions revealed significantly increased ACE after 72 h as compared to monocytes treated with NS. Further, HD serum potentiated the up-regulation of ACE by LPS (Fig. 1E, F).

### Uremia-mediated increase in ACE expression coincided with elevated adhesion and transmigration

In further investigations we tested whether uremic stimulus is able to affect the adhesion and transmigration of primary human monocytes and undifferentiated THP-1 cells. As shown in Fig. 1G, H primary monocytes reacted on HD stimulus with significantly increased number of the cells adhered to endothelial monolayers. Similarly, THP-1 treatment with HD for 72 h led to significantly stronger attachment abilities when compared to NS (Fig. 1I). Additionally HD-stimulus led to development of higher number of more differentiated cells than NS treatment (data not shown). Also under HD both cell-types transmigrated significantly faster through endothelial monolayers than corresponding controls (Fig. 1J, K, L, M).

**Figure 2:**
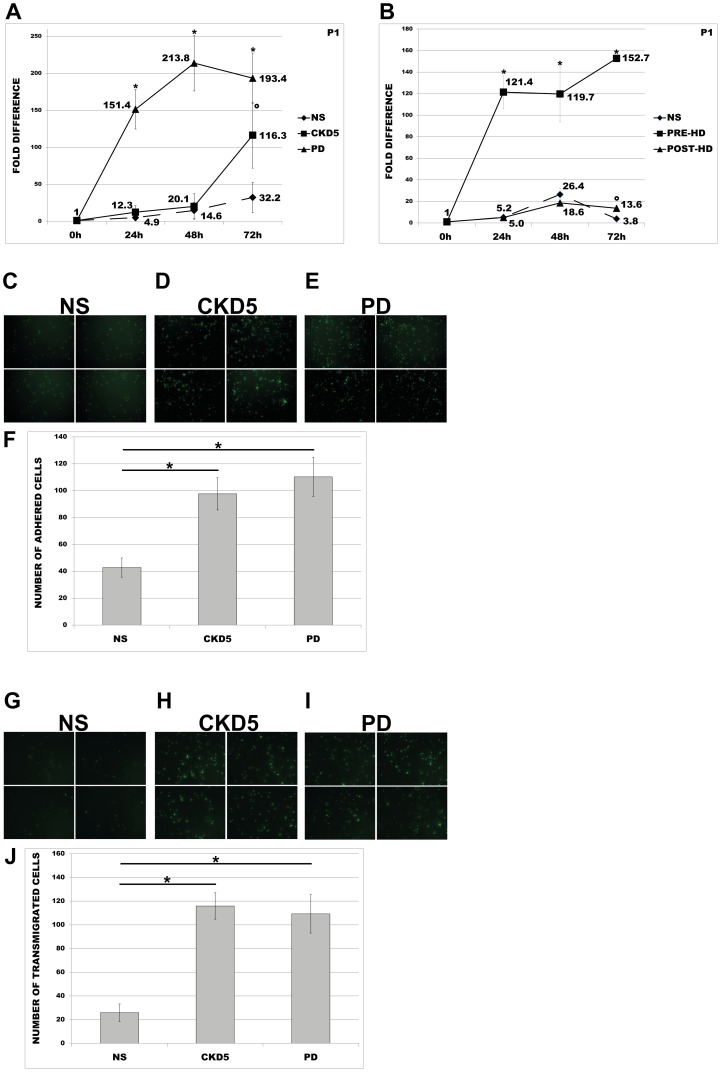
Expression of ACE and behaviour of human monocytes under uremic status II. (A, B) Primary human monocytes obtained from healthy volunteer P1 were treated with (A) 10% NS or PD or CKD5 or (B) 10% NS or pre- or post-HD sera for 72 h and investigated for ACE-mRNA expression. Means ± SD of three independent experiments. * p< 0.05 (PD vs. NS; pre-HD vs. NS; pre-HD vs. post-HD) and °p<0.05 (CKD5 vs. NS; post-HD vs. NS) indicate statistical significance. (C, D, E, F) Adhesion of primary monocytes treated with NS or PD or CKD5 sera. Primary human monocytes were incubated in the presence of 10% NS or PD or CKD5 sera for 30 min and investigated for their endothelial-adhesion. The number (F) and corresponding images of endothelial-adhered monocytes (C, D, E) are shown. Means ± SD of cell number in 10 microscopic fields in three independent experiments. (G, H, I, J) Transmigration of calcein-labelled primary monocytes through endothelial monolayer towards lower chamber filled with RPMI medium supplemented with 10% NS or PD or CKD5 sera. Transmigrated cells were visualized with fluorescent microscopy (G, H, I) and counted in 10 random fields (J). Representative images are shown. Means ± SD of cell number in 10 microscopic fields in three independent experiments. * p< 0.05 indicates statistical significance.

In order to test whether observed effects on primary monocytes may also be induced with sera coming from non-dialysed CKD patients or patients on other type of dialysis, we subjected P1-monocytes to the treatment with NS or PD or CKD5 sera. Additionally, to examine the pre- and post-dialysis effects of HD-sera on the regulation of ACE, the same cells were preconditioned in the presence of NS or sera obtained prior to (pre-HD) and after (post-HD) dialysis sessions. As demonstrated in Fig. 2A, treatment with PD or CKD5 sera for 72 h, exerted significantly up-regulated ACE expression pattern, similar to those observed under HD treatment. Furthermore, PD or CKD5 conditions led to significantly increased transmigration and endothelial adhesion of the monocytes when compared to corresponding controls (Fig. 2C, D, E, F, G, H, I, J). Treatment of the primary monocytes with pre- or post-HD sera for 72 h revealed significantly up-regulated ACE expression as compared to NS treatments. However, increase in ACE level in pre- HD treatments was significantly and noticeably higher as compared to post-HD or NS (Fig. 2B).

### Phenotype, attachment and adhesion of monocytes are affected by ACE

**Figure 3:**
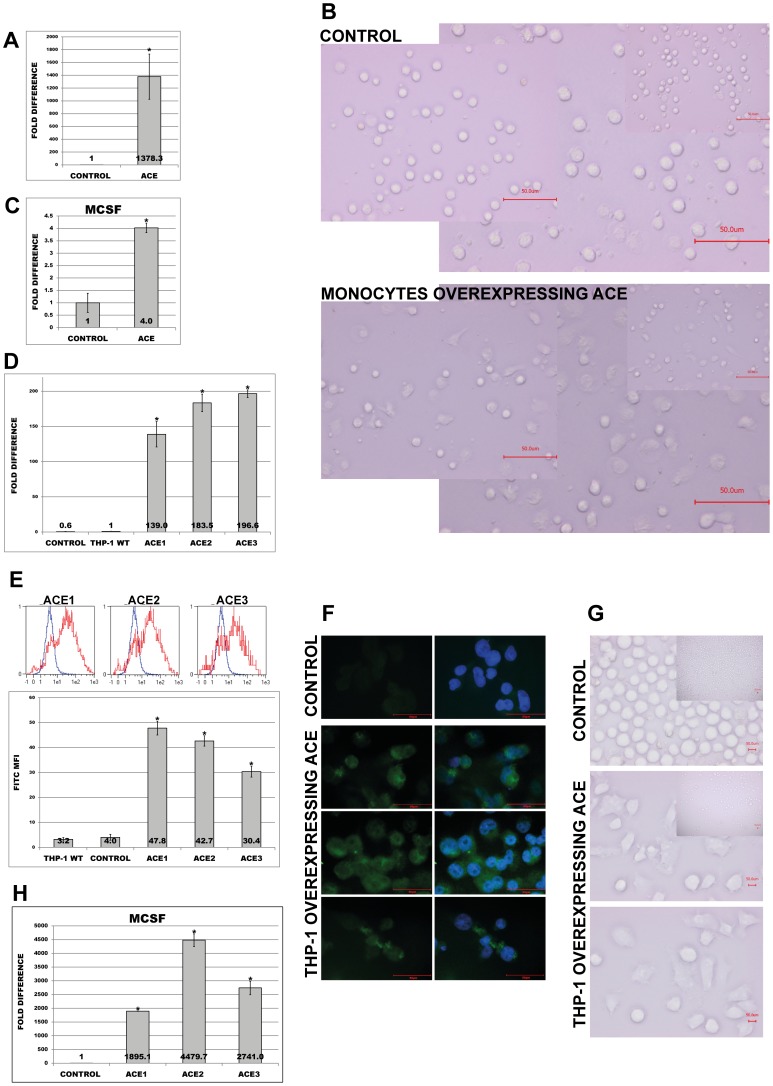
Expression of ACE and morphology of human monocytes overexpressing ACE. (A, B, C) Human primary monocytes were transiently transfected with empty or pcDNA3.1(-) plasmid carrying full coding sequence of ACE. Investigations of (A) ACE-expression, (B) cell morphology and (C) MCSF-expression were performed 24 h after transfection. Note differentiated, macrophage-like phenotype of primary monocytes overexpressing ACE. (D, E, F, G, H) Wild type (THP-1 WT), empty plasmid (Control) and ACE-overexpressing cells (ACE1, ACE2, ACE3) were investigated for (D) ACE transcript or (E) protein levels by employment of specific TaqMan probes or FACS analysis. (F) Representative immunofluorescence of control and ACE1 cells stained with FITC-ACE antibody (green) and DAPI staining (blue, nuclear). Note that left panel represents ACE staining only; right panel- ACE expression merged with DAPI; note mostly membrane-cytoplasmic localization of ACE. (G) Representative microscopic analysis of control and ACE1 cells under different magnifications. Note differentiated, macrophage-like phenotype of ACE1 cells. (H) RT-PCR analysis of MCSF expression in control and ACE-overexpressing cells (ACE1, ACE2, ACE3). Means ± SD of three independent experiments. * p< 0.05 indicates statistical significance.

To demonstrate a functional correlation between ACE expression and adhesive properties of the monocytes, we transfected primary human monocytes or THP-1 cells with plasmid carrying full coding sequence of human ACE. As demonstrated in Fig. 3A, transient transfection of primary monocytes resulted not only in significantly increased overexpression of ACE, but also led to development of more differentiated, macrophage-like phenotype (Fig. 3B). In addition these cells showed higher expression of MCSF (Fig. 3C).

**Figure 4:**
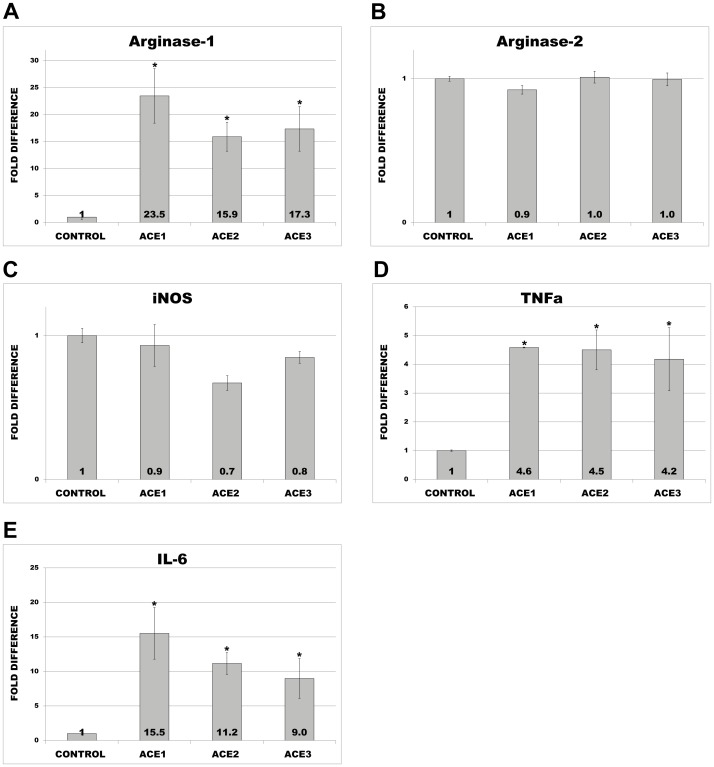
RT-PCR analysis of macrophage markers in control and THP-1 cells overexpressing ACE (ACE1, ACE2 and ACE3). Analyses were performed with primers specific for Arg1 (A), Arg2 (B), and iNOS (C), and TaqMan probes for TNFa (D) and IL6 (E). * p< 0.05 indicates statistical significance. Means ± SD of three independent experiments.

Due to the limited nature of transient transfection and very short life-span of primary monocytes, we generated THP-1 monocytes stably overexpressing ACE. For further experiments we selected three clones demonstrating the highest ACE levels (designated as ACE1, ACE2 and ACE3). Control cells were transfected with empty plasmid. The overexpression of ACE in THP-1 was verified by RT-PCR, FACS analysis and immunofluorescence microscopy (Fig. 3D, E, F). Similar to primary monocytes, ACE-clones revealed also a macrophage-like phenotype and significantly increased expression of MCSF (Fig. 3G, H). Further analysis of macrophage markers demonstrated noticeably elevated expression of Arg1; however no significant changes for Arg2 or iNOS were observed (Fig. 4A, B, C). Investigations of inflammation markers demonstrated a marked up-regulation of TNFa and IL-6 (Fig. 4D, E). These observations suggest that ACE may promote an alternative activation of the macrophages, leading to M2-phenotype with pro-inflammatory and pro-atherosclerotic properties.

**Figure 5:**
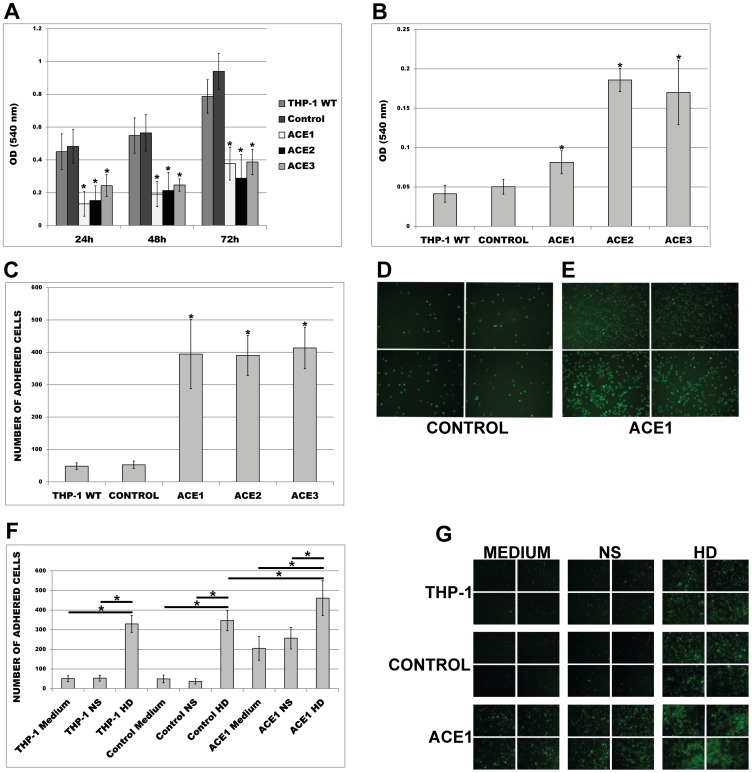
Proliferation, attachment and adhesion of ACE-overexpressing THP-1 monocytes investigated on wild type (THP-1 WT), empty plasmid (Control) and ACE-overexpressing cells (ACE1, ACE2, ACE3). (A) MTT-proliferation and (B) attachment of the cells. The cells for (B) were treated with 10 ng/ml PMA for 72 h prior to assay. (C, D, E, F, G) Adhesion of THP-1 to HUVEC endothelial monolayers. Calcein–labelled cells were incubated for 30 min in the presence of endothelial monolayers at the chamber bottom. Means ± SD of three independent experiments. (C) Adhesion under normal conditions (medium only, no FCS). See representative images (D, E). * p< 0.05 vs. control indicates statistical significance. (F) Adhesion under normal conditions or medium supplemented with 10% NS or HD sera. See representative images (G). * p< 0.05 indicates statistical significance. Note that for F and G one representative ACE-overexpressing clone (ACE1) was used. Analyses of (C, D, E, F, G) were performed in 10 random microscopic fields each. Means ± SD of cell number in 10 microscopic fields in three independent experiments.

Fig. 5A shows that these more differentiated, ACE-overexpressing cells revealed significantly decreased proliferation rates than corresponding controls. Furthermore, these cells demonstrated not only increased attachment to plastic surface (Fig. 5B), but also to endothelial human monolayers as compared to corresponding controls (Fig. 5C, D, E). To test whether uremia-mimicked conditions add to the transfection related effects we subjected wild type, empty plasmid and ACE1 cells to adhesion assays in the presence of HD or NS. As demonstrated in Fig. 5F, G treatment with HD not only led to significantly increased adhesion of control THP-1, but further amplified the adhesion of ACE1 cells.

### Overexpression of ACE led to increased transmigratory potential

**Figure 6:**
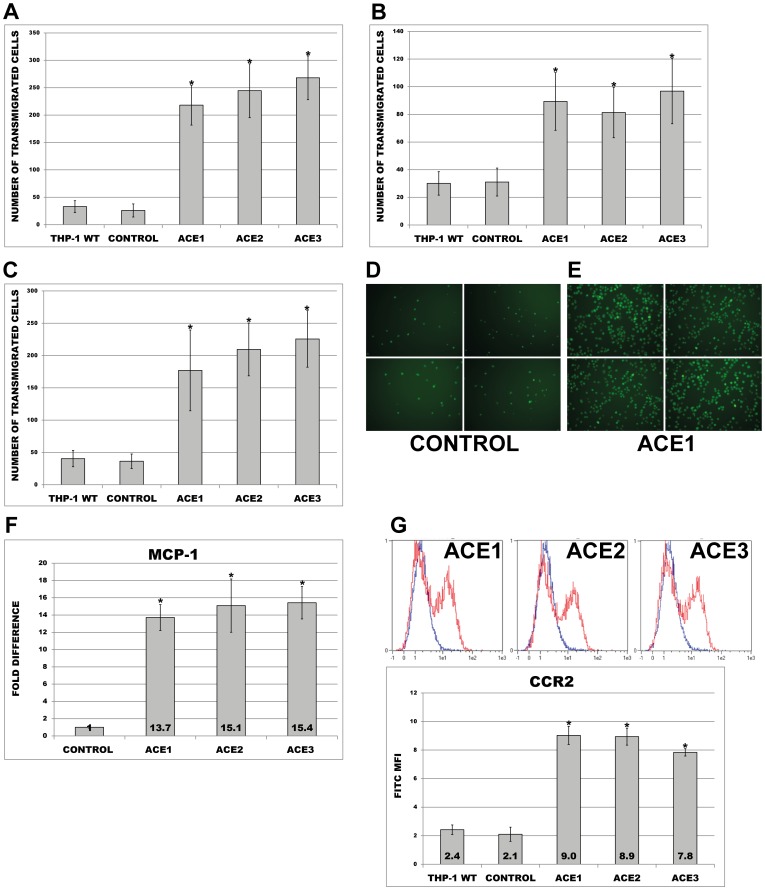
Transmigration of wild type (THP-1 WT), empty plasmid (Control) and ACE-overexpressing THP-1 monocytes (ACE1, ACE2, ACE3). (A) Transmigration of calcein-labelled cells through membrane towards (A) medium supplemented with MCP-1 or (B) HUVEC monolayers in the presence of medium only. (C, D, E) Transmigration of the cells through endothelial monolayers under MCP-1. See representative images (D, E). Analyses were performed in 10 random microscopic fields each. Means ± SD of cell number in 10 microscopic fields in three independent experiments. (F, G) Expression of MCP1 and CCR2 by RT-PCR and FACS analysis respectively. * p< 0.05 vs. control indicates statistical significance. Means ± SD of three independent experiments.

We observed that ACE-overexpressing cells possess stronger adhesion properties than corresponding controls. In order to investigate whether these cells are able to transmigrate faster through the endothelial barrier, we subjected wild type, control and ACE clones to transmigration assays. As demonstrated in Fig. 6A, the number of cells transmigrating through the membrane upon MCP-1 was significantly higher with ACE transfectants than corresponding controls. Significantly increased transmigratory potential of ACE clones was also demonstrated when the cells were transmigrated without MCP-1 towards an endothelial-monolayer seeded on the bottom of the chamber (Fig. 6B) or through an endothelial-barrier in the presence of MCP-1 (Fig. 6C, D, E).

We speculated that increased transmigration potential may be due to altered chemotactic signalling towards these cells. In order to prove this hypothesis, we investigated ACE-clones and control cells for the expression of MCP-1/CCL2 and its receptor CCR2. As demonstrated in Fig. 6F expression of MCP-1/CCL2 was noticeably increased in ACE-overexpressing cells than in empty plasmid cells and resulted in an up-regulation range of 13.7–15.4 fold. Similar results were obtained for CCR2 where FACS analysis revealed a 7.8–9.0 times increase in MFI as compared to corresponding control (Fig. 6G).

### Increased ACE levels affect the expression of adhesion-related genes and the status of Angiotensin II receptors

**Figure 7:**
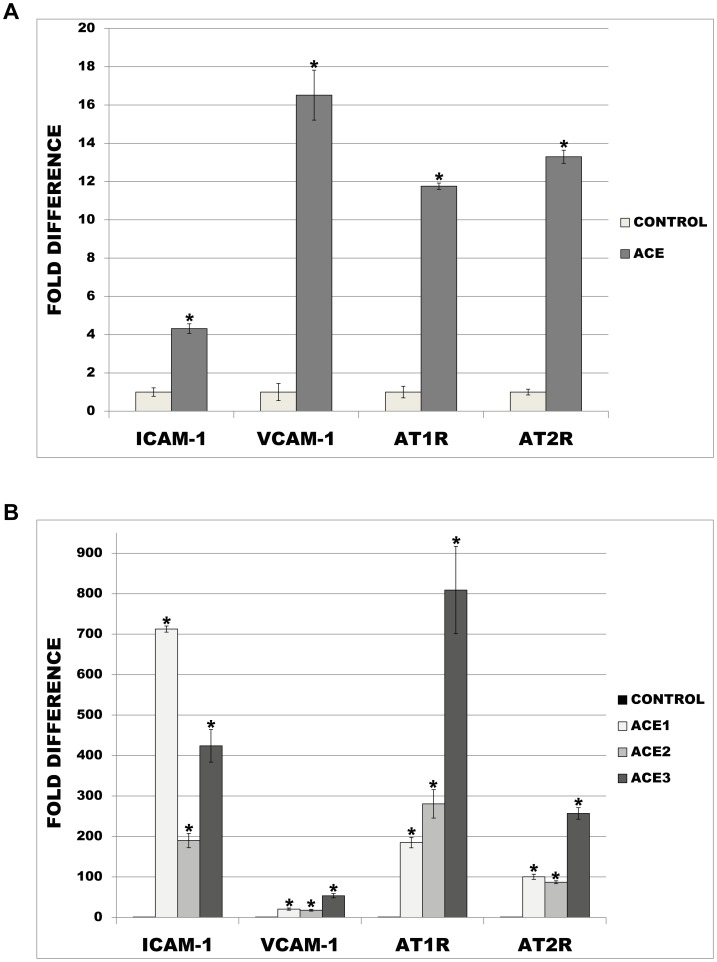
RT-PCR analysis of human primary monocytes and THP-1 cells overexpressing ACE. (A) Primary monocytes were transiently transfected with empty (Control) or ACE-plasmid and subjected to qPCR with primers specific for ICAM-1, VCAM-1, AT1R and AT2R. (B) Analysis of empty plasmid (Control) and ACE-overexpressing THP-1 monocytes (ACE1, ACE2, ACE3) were performed for the same transcripts. * p< 0.05 indicates statistical significance. Means ± SD of three independent experiments.

The effect of ACE-overexpression on adhesion-related genes was tested by qPCR. As demonstrated in Fig. 7A, expression of ICAM-1 and VCAM-1 was noticeably increased in primary monocytes transiently overexpressing ACE (4.3 and 16.5 fold increase respectively). ACE overexpression also led to a strong increase in the expression of AT1R (increase by factor 11.8) and AT2R (13.3). Investigations on THP-1 monocytes stably overexpressing ACE revealed similar expression patterns to those observed in primary monocytes (Fig. 7B). Expression of ICAM-1 and VCAM-1 was strongly up-regulated in all ACE-clones when compared to control cells (increase 189.9–712.6 fold and 17.8–53.5 fold respectively). Further ACE-mediated up-regulations were also observed for two AngII-receptors AT1R (increase by factor 184.8–809.2) and AT2R (86.9–257.0).

### Inhibition of ACE/AngII-receptor signalling and AT1R-blockage in AngII-treated THP-1 monocytes led to decreased adhesion to endothelial monolayers

**Figure 8:**
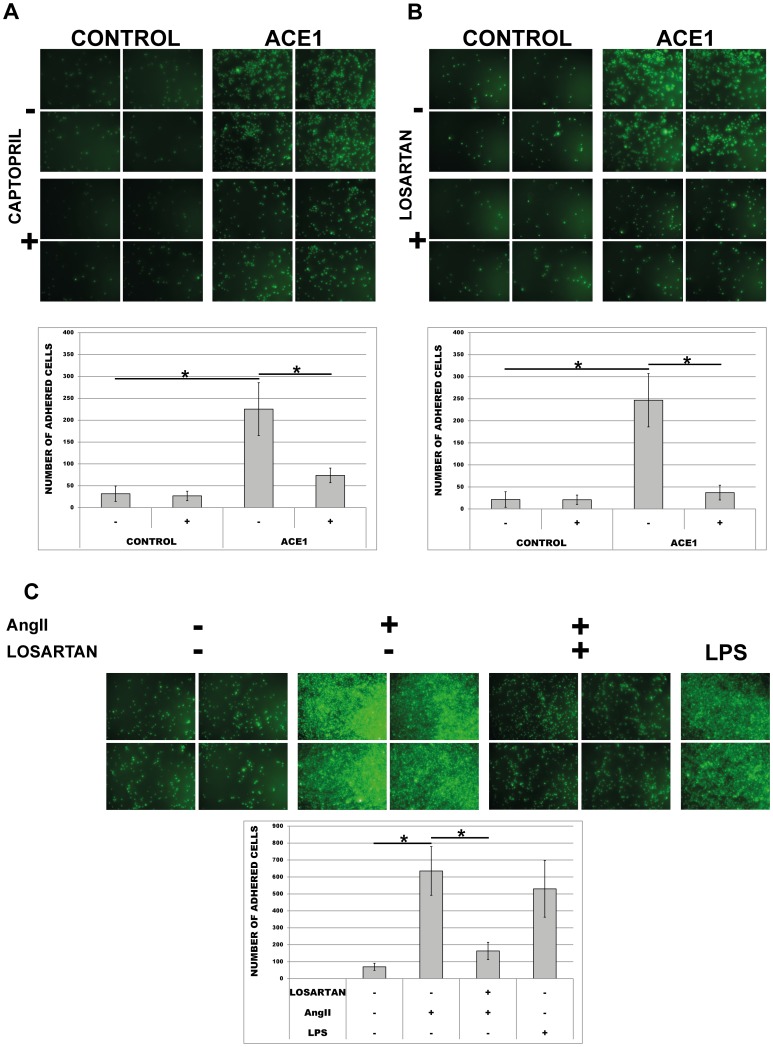
Effect of the ACE-inhibitor Captopril, the AngII-receptor blocker Losartan and AngII on adhesion of wild type (THP-1 WT), empty plasmid (Control) and ACE-overexpressing cells (ACE1). Calcein-labelled cells were incubated in the presence or absence of (A) 500 nM captopril or (B) 1 µM losartan for 30 min and tested for their adhesion abilities to endothelial HUVEC monolayers. (C) Endothelial-adhesion of ACE-negative wild type THP-1 cells in the presence of 1 µM AngII only or co-incubation with 1 µM losartan investigated for 30 min. Representative images for (A, B, C) are shown. Analyses for (A, B, C) were performed in 10 random microscopic fields each. * p< 0.05 indicates statistical significance. Means ± SD of cell number in 10 microscopic fields in three independent experiments.

To test whether inhibition of ACE/AngII-receptor signalling could affect the adhesive abilities of ACE-monocytes, the effects of ACE-inhibitor Captopril or AT1R-blocker Losartan were tested. As demonstrated in Fig. 8A, B, both drugs were effective in decreasing the endothelial adhesion of ACE-overexpressing monocytes significantly. These results suggest that higher endothelial-adhesion of ACE-overexpressing monocytes may be mediated by increased generation of local AngII and its receptor. To finally confirm these observations we subjected ACE-negative THP-1 wild type cells to adhesion assay with AngII and/or losartan. As demonstrated in Fig. 8C, adhesion of THP-1 is dramatically increased in the presence of AngII and almost totally abolished by AT1R-blockage (co-incubation with losartan).

## Discussion

This study clearly demonstrates a role of mACE for the uremia-associated atherogenic potential of monocytes and suggests an autocrine/paracrine mechanism of activation of these pro-atherogenic cells.

We found that both uremic and inflammatory conditions affect and regulate the expression of mACE. Our studies revealed that either treatment or prolonged monocyte differentiation in the presence of uremic or inflammatory conditions led to up-regulation of ACE. Furthermore, we were able to show that elevation of mACE coincided with increased transmigration and adhesion of these cells. To our knowledge, this is the first report demonstrating uremic elevation of mACE-expression in relation to pro-atherogenic behaviour of primary human monocytes or THP-1 cells. Such a potential of ACE was previously reported in animal and cell culture models but was not directly correlated with influences of uremia on human monocytes in vitro [26], [27], [28], [29]. With regards to other cell culture systems, it has been demonstrated that uremic toxins may affect oxidative burst activity of the leukocytes and increase their pro-inflammatory effects. This may contribute to the tendency to vascular damage in CKD [30]. Furthermore, Shimizu et al. reported that indoxyl sulphate (IS), one of the most representative uremic toxins, up-regulated AGT expression in proximal tubular cells [31]. In studies by Sun et al. IS and p-cresol sulphate up-regulated in addition to elevated AGT expression, other renin–angiotensin–aldosterone system (RAAS) components such as renin and AT1R [32]. Up-regulation of these RAAS components may subsequently exert, as observed in our study, increased expression of mACE and facilitate behavioural and morphological changes of the monocytes under uremia. Indeed, previous studies reported a possible link between uremic toxins and cardiovascular diseases. The authors demonstrated that uremia-mediated increase in leukocyte-endothelial adhesion occurs through elevation of E-selectin in HUVEC cells and is mediated via the JNK- and NF-κB-dependent pathway [33]. Furthermore, the studies by Vanholder et al. and Pletinck et al. showed clearly that proinflammatory effects exerted by protein-bound uremic toxins contribute to vascular damage and renal disease progression by stimulating crosstalk between leukocytes and vessels [34], [35]. On the other hand the existence of lipid or smooth muscle-derived serum factor which could be responsible for such alterations had been previously speculated [16], [36]. The levels of oxidized LDL are generally increased in hemodialysis patients and previous reports demonstrated that local AngII production increases as macrophages become activated by ox-LDL [37], [38]. Also the levels of MCP-1 and its receptor in uremic serum and atherosclerotic plaques were reported to be significantly higher than in healthy controls and could be the reason of increased transmigration [39].

What are the consequences of ACE-overexpression in human monocytes? Microscopic investigations revealed noticeable alterations in cell morphology. Introduction of ACE into monocytes changed not only their structure towards macrophage-like cells, but also dramatically elevated the expression of MCSF. These observations correlate with previous findings demonstrating that accumulation of monocyte-derived macrophages at the sites of endothelial dysfunction is a crucial event in atherogenesis [40]. Our data suggest that ACE mediates an alternative activation of macrophages and may promote M2-phenotype with pro-inflammatory and pro-atherosclerotic properties. ACE-overexpressing cells revealed not only significantly elevated levels of Arg1, but also pro-inflammatory cytokines TNFa and IL-6. It has been demonstrated that such M2 macrophages have a higher capacity to accumulate modified lipids than M1 and upon exposure to ox-LDL the pro-inflammatory responses of M2 cells are increased [41]. Furthermore, M2 cells are present in plaques where surround the lipid core. Arg1, typical for these cells, may promote stabilisation of atherosclerotic plaques and enhance the proliferation of vascular smooth muscle cells [42].

We demonstrated that ACE-overexpressing monocytes transmigrate through endothelial barrier significantly faster than corresponding controls as they express not only more MCP-1 but also its ligand CCR2. These novel findings are extremely important because the motility of these cells may be boosted in an autocrine manner independently from endothelial function. The fact that MCP-1 is up-regulated in atherosclerotic plaques and arteries of animals fed a high cholesterol diet and that disruption of CCR2 in mouse models is related to anti-atherosclerotic actions [43], [44], [45], [46], [47], allows us to designate ACE-overexpressing monocytes as highly pro-atherogenic.

Studies in mice revealed that infusion of AngII led not only to increased plaque size, but also induced the expression of inflammatory TNFa, IL-6, and migration-related MCP-1, CCR2 in aortic roots. In that study disruption of MCP-1 led to decrease in AngII-mediated pro-atherosclerotic events [48]. Interestingly, in CCR2 knock-out mice or mice bearing specific leukocyte CCR2-deficiency, AngII was not able to induce previously described vascular remodelling but promoted the development of left ventricular hypertrophy instead [49]. In studies by Chen at al. the authors demonstrated that ACE deficiency in bone marrow–derived cells decreased hypercholesterolemia-induced atherosclerosis and correlated with reduced levels of MCP-1[17].

ACE overexpression led to a marked up-regulation of ICAM-1 and VCAM-1 in monocytes. Expression and induction of these molecules has been consistently observed in the initial steps of atherosclerosis and in atherosclerotic plaques [50], [51]. Disruption or antibody-mediated blockage of these molecular targets proved to exert beneficial effects on atherogenesis [52], [53].

It is well documented that ACE-inhibition and/or anti-AngII-receptor treatment has anti-atherosclerotic effects in experimental models as well as patients with cardiovascular disease [54], [55], [56]. We found in our study that introduction of ACE into monocytes led to dramatically increased expression of AngII-receptors, AT1R and AT2R. ACE-inhibition or AngII-receptor blockage significantly decreased the adhesion of these monocytes to endothelial cells. These novel findings suggest that inhibition of local, monocyte-derived AngII-generation might exert anti-atherogenic actions.

Da Cunha et al. reported that subcutaneous infusions of AngII led to accelerated carotid atherosclerosis in apolipoprotein E-deficient mice. Additionally increased expression of adhesion molecules such as E-selectin, ICAM-1, VCAM-1, chemokine MCP-1, and MCSF was demonstrated. Enalapril, an ACE-inhibitor, reduced these expressions and the number of adhered macrophages and foam cells in the arterial wall [57].

We found that uremic serum on the one hand induces ACE overexpression, thus creating pro-atherogenic monocytes. In addition, uremic serum serves as an additional amplifier of monocyte-endothelial adhesion even for the cells already overexpressing ACE. This suggests that ACE induction is an important but most likely not the only mechanism by which uremia enhances monocyte endothelium interactions.

In summary we demonstrate uremia-induced elevation of ACE expression paralleled by a pro-atherogenic nature of ACE-overexpressing monocytes, partially mediated by enhancement of migratory and adhesion potential to endothelial monolayers. Inhibition of local, monocyte-derived AngII-generation exerts anti-atherosclerotic actions in vitro. These findings justify further investigation and verification in animal models.
